# From Preliminary Urinalysis to Decision Support: Machine Learning for UTI Prediction in Real-World Laboratory Data

**DOI:** 10.3390/jpm15050200

**Published:** 2025-05-16

**Authors:** Athanasia Sergounioti, Dimitrios Rigas, Vassilios Zoitopoulos, Dimitrios Kalles

**Affiliations:** 1 Department of Laboratory Medicine, General Hospital of Amfissa, 33100 Amfissa, Greece; 2 Independent Researcher, 33100 Amfissa, Greece; rigas_dimitris@yahoo.gr; 3 School of Science and Technology, Hellenic Open University, 26335 Patras, Greece; kalles@eap.gr

**Keywords:** urinary tract infection, machine learning, urinalysis, antimicrobial stewardship, threshold optimization, XGBoost, diagnostic decision support

## Abstract

**Background/Objectives**: Urinary tract infections (UTIs) are frequently diagnosed empirically, often leading to overtreatment and rising antimicrobial resistance. This study aimed to develop and evaluate machine learning (ML) models that predict urine culture outcomes using routine urinalysis and demographic data, supporting more targeted empirical antibiotic use. **Methods**: A real-world dataset comprising 8065 urinalysis records from a hospital laboratory was used to train five ensemble ML models, including random forest, XGBoost (eXtreme gradient boosting), extra trees, voting classifier, and stacking classifier. Models were developed using 10-fold stratified cross-validation and assessed via clinically relevant metrics including specificity, sensitivity, likelihood ratios, and diagnostic odds ratios (DORs). To enhance screening utility, threshold optimization was applied to the best-performing model (XGBoost) using the Youden index. **Results**: XGBoost and random forest demonstrated the most balanced diagnostic profiles (AUROC: 0.819 and 0.791, respectively), with DORs exceeding 21. The voting and stacking classifiers achieved the highest specificity (>95%) and positive likelihood ratios (>10) but exhibited lower sensitivity. Feature importance analysis identified positive nitrites, white blood cell count, and specific gravity as key predictors. Threshold tuning of XGBoost improved sensitivity from 70.2% to 87.9% and reduced false negatives by 82%, with an associated NPV of 96.4%. The adjusted model reduced overtreatment by 56% compared to empirical prescribing. **Conclusions**: ML models based on structured urinalysis and demographic data can support clinical decision-making for UTIs. While high-specificity models may reduce unnecessary antibiotic use, sensitivity trade-offs must be considered. Threshold-optimized XGBoost offers a clinically adaptable tool for empirical treatment decisions by improving sensitivity and reducing overtreatment, thus supporting the more personalized and judicious use of antibiotics.

## 1. Introduction

Urinary tract infections (UTIs) are among the most prevalent infections worldwide, posing a significant clinical and economic burden on healthcare systems [1]. They represent the most common outpatient infections, with at least half of all adult women experiencing a UTI during their lifetime. In healthcare settings, UTIs account for up to 9.4% of all hospital-acquired infections [2]. Despite advancements in diagnostics and treatment, UTIs continue to be associated with high morbidity and, in some cases, mortality [3].

Diagnosing UTIs remains challenging due to nonspecific symptoms and the delay in obtaining urine culture results. In emergency and primary care settings, clinicians often initiate empirical antibiotic treatment based on clinical suspicion to avoid delays in managing potentially severe infections [4]. However, this practice contributes to over-diagnosis and antibiotic overuse, contributing to the development of antimicrobial resistance. Previous studies suggest that 40–50% of patients empirically treated for UTIs may not have a true infection [5], and broader estimates suggest that nearly 28% of all antibiotic prescriptions—regardless of indication—may be clinically unnecessary [6].

Machine learning (ML) offers a data-driven approach to enhance diagnostic accuracy and support antimicrobial stewardship. Recent work has explored the ML-based prediction of urine culture outcomes using standard urinalysis data. For example, Parente et al. developed a random forest model to identify low-risk patients in primary care, potentially reducing unnecessary antibiotic use [7]. Dedeene et al. proposed an artificial intelligence support tool for rapid prediction of culture results in emergency settings [8], while Seheult et al. implemented a decision tree model for optimizing urinalysis-based UTI prediction [9].

Additional studies have explored related approaches, including the prediction of recurrent UTIs in pediatric populations using ML algorithms [10] and the development of interpretable models for critical UTI outcomes [11]. Choi et al. proposed an AI-based prediction framework for both urinary tract infections and associated bloodstream infections, supporting the early identification of high-risk patients [12]. Recent advances have also emphasized the importance of explainability in model development, facilitating clinical trust and adoption [13]. In a recent conceptual model, point-of-care diagnostic strategies for UTIs were shown to support antimicrobial stewardship by reducing empirical treatment in low-risk patients [14]. Recent findings suggest that the predictive performance of urinalysis may vary depending on the specific causative microorganism, indicating a need for microorganism-aware modeling strategies [15].

These advances highlight the potential of machine learning to improve diagnostic decision-making in UTI care. The present study aims to evaluate and compare the performance of five supervised ensemble ML models in predicting UTIs based on urinalysis and demographic features. Emphasis is placed on diagnostic metrics with clinical relevance—including specificity, the positive predictive value (PPV), and the positive likelihood ratio (PLR)—with the goal of supporting more rational empirical antibiotic use.

The key contributions of this study include (i) the development of an interpretable ML pipeline using real-world laboratory data; (ii) a comparative performance analysis of five ensemble classifiers; and (iii) a proposal of high-specificity models as potential tools to improve the management of empirical antibiotic prescriptions in suspected UTI cases, thereby supporting the implementation of a personalized approach in managing UTIs.

Although machine learning applications to urinary tract infection (UTI) diagnosis have been reported in the previous literature [7,8,9,12], most studies rely on EHR-based variables or clinical symptoms. By contrast, our study focuses exclusively on laboratory data, which enhances generalizability and simplifies integration into LISs. Furthermore, the application of both soft voting and stacking classifiers to real-world UTI data remains underexplored, particularly in low-prevalence populations. These aspects constitute important methodological distinctions and potential contributions to the field.

## 2. Materials and Methods

### 2.1. Dataset Description

The dataset used in this study was collected from the Laboratory Medicine Department of the General Hospital of Amfissa, Greece. It comprised urinalysis parameters, urine culture results, and demographic information from patients who underwent routine urinalysis and urine culture. Urinalysis data were obtained using the iChemVELOCITY Automated Urinalysis Analyzer (Leriva Diagnostics, Athens, Greece), an automated laboratory instrument employed in routine clinical practice. The analyzer evaluates urine dipstick tests using reflectance photometry and standardized colorimetric methods, providing operator-independent measurements of physical and chemical urine characteristics. The reported parameters included specific gravity (SG), pH, color, clarity, ketones, glucose, protein, nitrites, bilirubin, urobilinogen, white blood cells (WBCs), and blood. All results were retrospectively extracted from the laboratory information system and fully de-identified prior to analysis. Urine culture results were classified as positive when bacterial growth exceeded 10^5^ cfu/mL and as non-positive otherwise, in accordance with established microbial threshold guidelines [16,17,18].

The dataset included 8065 urine samples, of which 2257 (28.0%) were classified as positive and 5808 (72.0%) as non-positive. Of the total samples, 3111 (38.6%) were from male and 4954 (61.4%) from female patients. The mean patient age was 57.8 years (SD = 25.0). Among males, 22.6% of cultures were positive, while the positivity rate among females was 29.1%.

### 2.2. Data Preprocessing

• Missing Data Handling: Only participants with complete laboratory data were included in the final analysis. We deliberately avoided imputation to ensure methodological transparency and to rely exclusively on observed values. This decision supports internal validity by avoiding bias introduced through statistical assumptions. While this approach reduced the available sample size, it ensured that all diagnostic metrics reflect true laboratory distributions. Importantly, even if imputation had been applied, the number of false negative cases in the dataset would still have remained insufficient for the robust training of cost-sensitive models or subgroup analyses.
• Feature Engineering and Encoding: Categorical variables (gender, color, and clarity) were encoded using one-hot or ordinal encoding, depending on their characteristics. Numerical variables were standardized where appropriate [19]. All available variables were retained across models to ensure comparability, and no dimensionality reduction techniques were applied.
• Class Imbalance Handling: Although the proportion of positive cultures (26.2%) did not constitute severe imbalance, it was sufficient to potentially bias model learning. Initial attempts using synthetic oversampling techniques such as SMOTE and ADASYN degraded precision and increased false positives due to noisy synthetic samples. Therefore, all models were ultimately trained using a balanced bagging classifier framework. This ensemble-based resampling method improves minority class representation while preserving the original data structure and avoiding synthetic artifacts. Its reliability in structured clinical datasets has been previously demonstrated [20,21,22].

### 2.3. Model Development

This study focused on five ensemble learning algorithms, selected for their robustness, generalization capacity, and proven performance in clinical tabular data, namely random forest, XGBoost, extra trees, voting classifier, and stacking classifier. Each model was developed using 10-fold stratified cross-validation to ensure balanced representation of positive and negative cases across training and validation subsets. All preprocessing steps—including encoding and scaling—were embedded within scikit-learn pipelines to prevent data leakage [23,24,25,26].

The random forest and extra trees classifiers are both ensembles of decision trees but differ in how splits are determined; extra trees uses full randomization to reduce variance and is often less prone to overfitting in imbalanced settings. XGBoost, a gradient-boosted decision tree algorithm optimized for predictive accuracy, was fine-tuned using randomized grid search over key hyperparameters such as the learning rate, maximum depth, and number of estimators [27,28].

The voting classifier aggregates predictions from random forest, XGBoost, and extra trees using soft voting, averaging the predicted class probabilities to determine the final outcome. The stacking classifier employs the same base learners, with logistic regression as the meta-learner to generate a final calibrated prediction. This setup offers the advantage of combining model diversity with interpretable outputs [29,30,31,32].

All models were implemented using the scikit-learn and XGBoost libraries in Python 3.10. Preprocessing and model training were structured using scikit-learn’s Pipeline and ColumnTransformer objects. The ColumnTransformer allowed for the application of different preprocessing steps to numeric and categorical features, whereas Pipeline combined preprocessing and modeling into a single, cross-validation-safe workflow, preventing information leakage and ensuring reproducibility.

### 2.4. Evaluation Metrics

Performance was evaluated using a set of standard diagnostic metrics, selected to provide a structured and clinically relevant assessment of model performance:

• Accuracy: Proportion of correct predictions (true positives and true negatives) among all evaluated cases.
• Balanced Accuracy: Mean of sensitivity and specificity. Suitable for imbalanced datasets, as it considers both classes equally.
• Sensitivity (Recall): Proportion of actual positive cases correctly identified by the model.
• Specificity: Proportion of actual negative cases correctly identified. High specificity reduces the likelihood of false positives.
• Precision (Positive Predictive Value—PPV): Proportion of predicted positive cases that are true positives.
• Negative Predictive Value (NPV): Proportion of predicted negative cases that are true negatives.
• F1 Score: Harmonic mean of precision and recall. Useful when both false positives and false negatives carry clinical consequences.
• ROC AUC: Area under the receiver operating characteristic curve. Reflects overall discrimination capacity of the model across thresholds.
• Matthews Correlation Coefficient (MCC): A balanced measure that incorporates all components of the confusion matrix. Appropriate for imbalanced datasets.
• Positive Likelihood Ratio (PLR): How much more likely a positive test result is in someone with the condition than in someone without it. Values > 10 are considered strong evidence to support a diagnosis.
• Negative Likelihood Ratio (NLR): Ratio of the false negative rate to the true negative rate. Lower values suggest the test is effective at excluding disease; values < 0.1 are generally considered acceptable.
• Diagnostic Odds Ratio (DOR): The ratio of the odds of a positive test result in patients with the disease to the odds of the same result in those without it. It integrates sensitivity and specificity into a single indicator of discriminative power [33,34,35].

### 2.5. Model Interpretation and Performance Visualization

To assess model interpretability, we applied permutation feature importance (PFI), a model-agnostic approach that quantifies the contribution of each predictor by measuring the change in model performance when the values of that feature are randomly shuffled. This method was implemented using the permutation importance function from scikit-learn, applied to cross-validated predictions to ensure robustness [36,37].

In addition to an importance analysis, ROC curves, precision–recall curves, and confusion matrices were generated to support a graphical evaluation of diagnostic performance. These plots were created using predicted probabilities and model outputs aggregated across all cross-validation folds. Interpretation and visualization focused primarily on the top-performing models, as identified by their high specificity, PPV, and positive likelihood ratios.

### 2.6. Threshold Optimization Procedure

Among the evaluated classifiers, the XGBoost model demonstrated the most clinically balanced performance in terms of diagnostic accuracy, interpretability, and generalization. To further improve its clinical sensitivity, a post hoc threshold optimization procedure was applied to the pooled validation predictions. This process penalized false negatives more heavily than false positives and used a custom cost function to reflect clinical priorities. The optimal probability cutoff was selected using the scipy.optimize.minimize_scalar method, which minimized the cost function over a continuous range of thresholds. For comparison, we also computed the Youden index [38], but it was not selected as the final decision criterion. This post-training adjustment aimed to enhance the model’s ability to detect true positive cases without excessively compromising specificity.

## 3. Results

The comparative performance of all models is summarized in Table 1. While the voting and stacking classifiers achieved the highest specificity (95.6% and 95.4%, respectively) and positive likelihood ratios (PLR > 10), their sensitivity was notably low, indicating limited ability to detect all true positive cases. These characteristics suggest potential utility in scenarios where false positives must be minimized but raise concerns regarding their suitability for general screening purposes.

In contrast, the XGBoost classifier exhibited the most balanced overall profile. Combining high specificity with strong overall discriminative performance, it emerged as a versatile model for clinical settings—supporting the exclusion of culture-negative cases without disproportionately compromising the detection of true infections. The random forest classifier showed similar behavior, with a closely aligned diagnostic odds ratio and performance trade-offs. These two models offered a more even distribution of diagnostic strengths, suggesting their greater suitability for scenarios where both false positives and false negatives carry clinical implications.

The extra trees classifier also performed well in terms of specificity and the positive likelihood ratio, although its overall detection capacity appeared more limited. This places it between the high-specificity models and those offering broader balance across diagnostic metrics.

Across all classifiers, the negative predictive value (NPV) remained high (>88%), suggesting reliability in identifying patients without UTIs. However, none of the models achieved an NLR below 0.1, a threshold typically required for effective rule-out tools. As such, while models like XGBoost may assist in reducing unnecessary empirical treatment, they should be interpreted cautiously and within a broader clinical decision-making framework.

For all models, feature importance was assessed using permutation importance (PFI) based on accuracy loss. Feature ranking was visualized using bar plots, and consistent key predictors across models included WBC count, protein, and urine pH.

**Figure 1:**
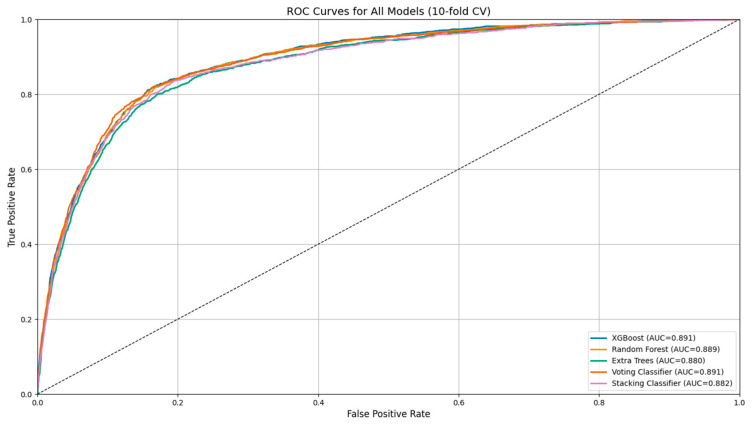
ROC curves for all models based on 10-fold cross-validation. All classifiers demonstrated comparable AUC values, with XGBoost achieving the highest value (AUC = 0.892).

**Figure 2:**
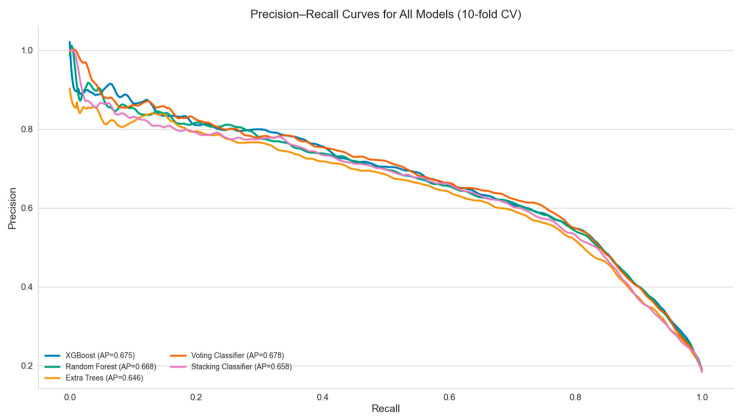
Precision–recall curves for all models based on 10-fold stratified cross-validation. Despite their higher average precision, the voting and stacking classifiers exhibited markedly reduced recall, limiting their use in general screening.

**Figure 3:**
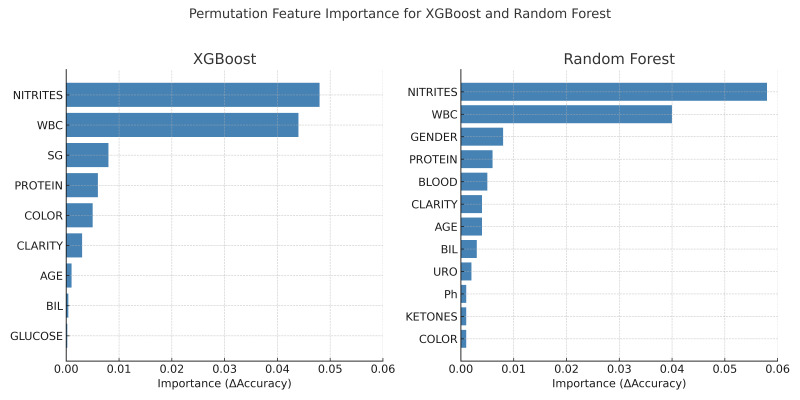
Permutation feature importance for the XGBoost and random forest classifiers. Importance scores represent the mean decrease in classification accuracy when each feature is permuted. Nitrites and white blood cell (WBC) count were the most influential features across both models.

In addition to importance analysis, ROC curves (Figure 1) and precision–recall curves (Figure 2) were generated to support the graphical evaluation of diagnostic performance. These plots were created using predicted probabilities and model outputs aggregated across all cross-validation folds. To further enhance model interpretability, permutation feature importance (PFI) was applied to the XGBoost and random forest classifiers. As illustrated in Figure 3, both models consistently identified positive nitrites (NITRITES_POSITIVE), WBC count, and specific gravity (SG) as the most informative predictors for UTI diagnosis.

Among the five evaluated ensemble models, XGBoost demonstrated the highest F1 score (0.648) and ROC AUC (0.887), as well as a favorable diagnostic odds ratio (DOR: 20.52). While random forest showed similar performance, XGBoost consistently achieved marginally superior results across multiple clinically relevant metrics, including balanced accuracy, specificity, and Matthews correlation coefficient. These findings, along with its capacity for fine-tuning through hyperparameter and threshold optimization, supported its selection for post hoc enhancement.

To improve clinical sensitivity, the XGBoost model underwent threshold tuning using a probabilistic cutoff derived from the Youden index, following a similar approach to that described in previous machine learning studies for diagnostic model optimization [39,40].

**Figure 4:**
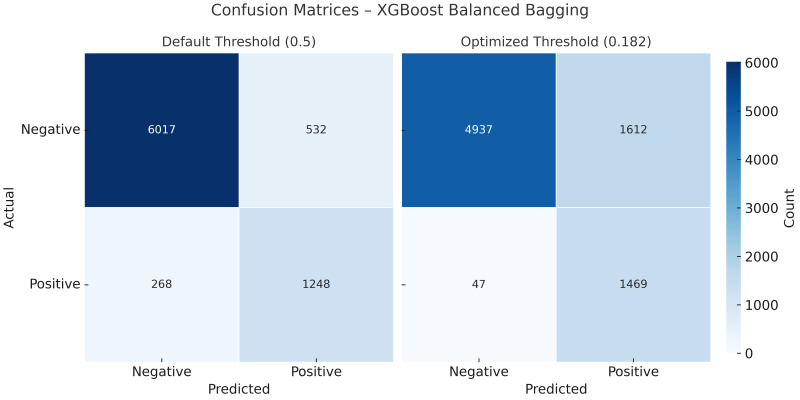
Confusion matrices for the XGBoost classifier before (**left**) and after (**right**) probability threshold optimization (cutoff = 0.182). The optimized threshold reduced false negatives while increasing true positives, sensitivity, and NPV at the expense of lower specificity and precision.

Figure 4 shows the comparison of confusion matrices before and after threshold optimization. Applying the optimized threshold of 0.182 resulted in a marked reduction in false negatives (268 → 47), with a corresponding increase in true positives (1248 → 1469). This adjustment increased sensitivity from 70.2% to 87.9% and improved the negative predictive value (NPV) from 92.8% to 96.4%. However, this improvement came at the expense of increased false positives (532 → 1612), leading to a reduction in specificity (89.6% → 74.1%) and precision (60.9% → 44.1%). These changes reflect the expected trade-off between recall and precision, particularly relevant in screening contexts where missing infections may carry significant risk. ROC and precision–recall curves remained unchanged, as threshold tuning only alters classification decisions without affecting the predicted probabilities.

The complete set of performance metrics before and after threshold optimization is summarized in Table 2. The results illustrate the expected trade-off introduced by post hoc adjustment: the sensitivity, negative predictive value, and diagnostic odds ratio were substantially improved, while specificity and precision declined. Notably, the ROC AUC remained unchanged, as threshold tuning does not alter predicted probabilities.

## 4. Discussion

### 4.1. Overview of Findings

This study evaluated five ensemble machine learning models for predicting urinary tract infections (UTIs) based on demographic and urinalysis data. Rather than focusing solely on accuracy, the analysis emphasized clinically meaningful metrics such as specificity, likelihood ratios, and predictive values to assess each model’s potential for real-world diagnostic support.

Among the evaluated models, XGBoost and random forest exhibited the most balanced diagnostic profiles, achieving diagnostic odds ratios (DORs) of 21.20 and 21.65, respectively, along with the highest balanced accuracy values. These findings suggest that both models may offer dependable support in identifying patients at risk for UTIs.

By contrast, the voting and stacking classifiers achieved the highest specificity (>95%) and positive likelihood ratios (>10), traditionally associated with ruling-in disease. However, their markedly lower sensitivity reduces their suitability for general screening or settings where under-treatment carries significant clinical risk.

### 4.2. Diagnostic Implications

Although none of the models achieved a negative likelihood ratio (NLR) below the conventional 0.1 threshold required for reliable clinical rule-out [41,42], most exhibited acceptable-to-high negative predictive values (NPVs), particularly XGBoost (92.8%) and random forest (93.4%). These values suggest potential utility in supporting decisions to withhold empirical antibiotics in low-risk scenarios. However, for models with NPVs below 90%, such as the stacking and voting classifiers (88.7% and 88.9%, respectively), clinical caution is warranted.

The limited sensitivity observed—especially in the high-specificity classifiers—is likely attributable not to algorithmic limitations but to the restricted clinical context available in the dataset. In the absence of symptomatology (e.g., fever, dysuria, or urgency), the models rely exclusively on urinalysis and demographic data, which may not capture the full clinical spectrum of UTI presentations. This highlights the importance of incorporating richer clinical features when higher recall is desired.

Previous research by Ourani et al. showed that using predefined reflex-to-culture criteria based on urinalysis could safely reduce unnecessary antibiotic prescriptions, supporting the idea that properly tuned ML tools may complement such clinical pathways [14].

XGBoost achieved the highest F1 score and ROC AUC among all models, indicating a favorable balance between case detection and diagnostic precision. Random forest performed comparably. While extra trees also demonstrated strong specificity, its lower sensitivity resulted in a less favorable diagnostic trade-off.

### 4.3. Impact of Threshold Optimization on Diagnostic Performance

While the XGBoost classifier demonstrated the most balanced performance among the evaluated models, its default sensitivity was not sufficient for reliable case detection in screening contexts. To address this, we applied post hoc threshold optimization using the Youden index. Instead of retraining the model, we adjusted the decision threshold based on probabilistic outputs to improve the identification of true positives.

This strategy markedly reduced false negatives and increased sensitivity, offering a clinically favorable trade-off despite the associated decrease in specificity and precision. Importantly, model discrimination—as measured by the ROC AUC—remained unchanged, confirming that performance improvements were attributable solely to threshold adjustment rather than overfitting or model instability.

From a clinical perspective, threshold tuning offers a simple yet effective mechanism for tailoring model behavior to specific use cases, such as prioritizing early infection detection. This flexibility is particularly valuable in operational environments where retraining is not feasible, allowing clinicians and informatics teams to adapt model outputs to evolving clinical priorities. As such, threshold optimization can serve as a pragmatic bridge between model development and real-world deployment, especially in interdisciplinary workflows involving both data scientists and healthcare providers.

### 4.4. Model Interpretability and Clinical Integration

Permutation feature importance (PFI) analysis revealed consistent key predictors across models, notably the positive nitrites test, WBC count, and SG. These features align with established clinical markers of UTIs and enhance the transparency of model decisions, supporting integration into decision support tools. These findings mirror those reported by Chambliss et al., who demonstrated strong correlation between specific chemical urinalysis elements and positive cultures, highlighting the potential of automated workflows in initial infection screening [41].

All models were trained solely on structured, non-microscopic laboratory parameters—such as color, clarity, WBCs, nitrites, and pH—along with demographic variables including age and gender. This configuration facilitates real-time integration into laboratory information systems (LISs) and electronic health records (EHRs), supporting clinical decision-making in primary care settings and emergency departments.

Although the voting classifier achieved excellent specificity and a high positive likelihood ratio, its limited sensitivity restricts its usefulness in screening applications, where reliably identifying true cases is a central concern. Nevertheless, it may serve a valuable role in antimicrobial stewardship strategies aimed at reducing unnecessary antibiotic prescriptions. In our dataset, deploying such a high-specificity model could reduce empirical antibiotic use from a baseline of approximately 45% to just 10–11% of patients, representing a substantial step toward more judicious prescriptions.

Beyond its diagnostic improvements, the threshold-optimized XGBoost model offers substantial advantages in clinical antimicrobial stewardship. In typical empirical practice, up to 45% of patients with nonspecific urinalysis findings may receive antibiotics without confirmed infection, leading to overtreatment and potential antimicrobial resistance. In contrast, our model recommended treatment for only 38.2% of cases, and just 19.99% of the total population would have received unnecessary antibiotics (i.e., false positives). This corresponds to an absolute reduction of 25 percentage points and a relative reduction of approximately 56% in overtreatment compared to empirical prescriptions.

In clinical practice, treatment decisions for suspected UTIs must almost always be made prior to the availability of culture results, as standard urine culture remains a time-consuming process. Although rapid molecular platforms (e.g., PCR-based diagnostics) are emerging, their real-world application remains in its early stages. Significant time and clinical experience will be required to evaluate their performance and determine whether they can be effectively adapted to meet the diagnostic needs of frontline physicians [43,44,45,46,47]. Additionally, their current limitations in availability, high cost, and limited clinical validation further restrict their widespread adoption.

In this context, machine learning–based decision support systems offer a practical and scalable alternative for improving diagnostic accuracy and guiding antibiotic prescribing—particularly in care environments where empirical decisions must be made before definitive culture results become available.

### 4.5. Limitations

This study is subject to several limitations. First, it was conducted using retrospective data from a single institution, which may limit generalizability across diverse populations or care settings. Second, the models relied exclusively on laboratory and demographic data without incorporating clinical presentation, comorbidities, symptom duration, or prior antibiotic use—factors that can influence infection likelihood and diagnostic interpretation.

Although stratified 10-fold cross-validation was applied to minimize overfitting, external validation on independent datasets is essential before considering clinical deployment. Additionally, moderate class imbalance and the exclusion of incomplete records may have introduced bias or reduced model robustness.

Despite these limitations, this study presents a reproducible and interpretable machine learning framework using real-world urinalysis data. The results provide a foundation for the future development of decision support tools and highlight the potential value of integrating predictive modeling into clinical laboratory workflows. Nevertheless, future work should include external validation across multiple institutions to account for differences in laboratory protocols, patient demographics, and diagnostic pathways.

### 4.6. Future Directions

Looking ahead, future research should focus on incorporating structured clinical features—such as urinary symptoms, comorbidities, and prior infection history—to enhance sensitivity and better capture the full clinical spectrum of UTI presentations. Prospective validation across diverse healthcare environments, including primary care and emergency departments, is essential to assess the generalizability and real-world impact of these models.

Moreover, the development of hybrid diagnostic frameworks that combine ML predictions with structured clinical criteria (e.g., dysuria, fever) or symptom-based rules may offer improved interpretability and greater clinical acceptance. Investigating the cost-effectiveness of such tools and their influence on prescription behavior and antimicrobial resistance trends is a critical step toward sustainable clinical deployment. Finally, advances in explainable artificial intelligence (XAI) and user-centered interface design will be crucial for fostering trust and facilitating integration into laboratory information systems and electronic health records.

It is essential to emphasize that such tools are intended solely to complement—not replace—clinical judgment. The treating physician remains solely responsible for diagnosis and patient management, and these models should only be applied within clearly defined clinical pathways that preserve the primacy of medical decision-making.

### 4.7. Clinical Applicability

While the present study focuses on model development using retrospective, single-site laboratory data, we acknowledge the importance of future integration into clinical workflows. In accordance with TRIPOD guidelines, such integration requires robust external validation, calibration, and contextual adaptation. Moreover, deployment should take into account institutional variability in laboratory practices and diagnostic workflows. Potential applications of the proposed model include early alert systems within laboratory information systems (LISs), support for empirical antimicrobial therapy in emergency settings, and the prioritization of diagnostic testing in high-risk patients. These scenarios highlight the potential for practical use but warrant prospective evaluation before implementation.

## 5. Conclusions

This study demonstrates that machine learning models based on routine urinalysis and demographic data can support clinical decision-making in suspected urinary tract infections. Among the evaluated classifiers, XGBoost and random forest exhibited the most balanced diagnostic profiles, combining high specificity with interpretability and overall discriminative capacity.

The voting classifier achieved the highest specificity and positive likelihood ratio, suggesting potential utility in reducing unnecessary antibiotic prescriptions. However, the limited sensitivity observed across all models restricts their use as independent diagnostic tools, particularly in clinical settings, where failure to detect infections may have consequences.

Given the absence of symptom-based clinical features in the dataset, we opted for post hoc threshold optimization rather than retraining in order to realign the XGBoost model with practical clinical needs. This adjustment achieved a more favorable balance between sensitivity and specificity, reducing false negatives while also lowering overtreatment rates compared to empirical prescriptions.

These models—particularly XGBoost—may assist in identifying low-risk patients and guiding empirical treatment decisions in resource-constrained or high-volume settings. Their strength lies in stratifying patients according to infection risk using readily available structured data, thereby supporting antimicrobial stewardship and advancing individualized care.

Future research should focus on external validation in diverse populations, the incorporation of clinical variables such as symptoms and comorbidities, and implementation studies assessing real-world impact. It is essential to maintain clear boundaries between algorithmic support and clinical responsibility; these tools are designed to inform but not substitute the diagnostic judgment and accountability of licensed healthcare professionals.

## Figures and Tables

**Table 1:** The detailed results, including accuracy, sensitivity, specificity, PPV, NPV, F1 score, balanced accuracy, PLR, NLR, and DOR for each classifier.

Model	Accuracy	Balanced Accuracy	Precision (PPV)	Recall (Sensitivity)	Specificity	NPV	F1 Score	ROC AUC	MCC	PLR	NLR	DOR
Random Forest	0.856	0.808	0.596	0.731	0.885	0.934	0.657	0.888	0.572	6.42	0.30	21.65
XGBoost	0.859	0.799	0.610	0.702	0.896	0.928	0.670	0.892	0.575	6.79	0.33	21.20
Extra Trees	0.857	0.756	0.626	0.595	0.918	0.918	0.655	0.888	0.571	7.34	0.44	17.23
Stacking Classifier	0.864	0.714	0.706	0.473	0.954	0.887	0.566	0.883	0.503	10.69	0.552	19.77
Voting Classifier	0.868	0.721	0.717	0.486	0.956	0.889	0.579	0.891	0.518	11.29	0.538	21.63

Table 1: Diagnostic performance metrics of five ensemble classifiers for UTI prediction using urinalysis and demographic features.

**Table 2:** Comparative performance of the XGBoost model before and after post hoc threshold optimization (cutoff = 0.182).

Metric	XGBoost (Default Threshold)	XGBoost (Optimized Threshold)
Accuracy	0.859	0.767
Balanced Accuracy	0.799	0.810
Precision (PPV)	0.609	0.441
Recall (Sensitivity)	0.702	0.879
Specificity	0.896	0.741
Negative Predictive Value (NPV)	0.928	0.964
F1 Score	0.652	0.587
ROC AUC	0.886	0.886
Matthews Corr. Coef. (MCC)	0.567	0.501
Positive Likelihood Ratio (PLR)	6.740	3.401
Negative Likelihood Ratio (NLR)	0.333	0.163
Diagnostic Odds Ratio (DOR)	20.250	20.893

## Data Availability

The data presented in this study are not publicly available due to privacy and ethical restrictions, as they contain sensitive patient information collected from a clinical laboratory setting. Access to the dataset is restricted by the institutional policies of the General Hospital of Amfissa and cannot be shared publicly.

## Abbreviations

The following abbreviations are used in this manuscript:

UTIs	Urinary Tract Infections
ML	Machine Learning
SG	Specific Gravity
WBC	White Blood Cell
SMOTE	Synthetic Minority Over-sampling Technique
ADASYN	Adaptive Synthetic Sampling
XGBOOST	eXtreme Gradient Boosting
PPV	Positive Predictive Value
NPV	Negative Predictive Value
ROC-AUC	Receiver Operating Characteristic Area Under the Curve
MCC	Matthews Correlation Coefficient
PLR	Positive Likelihood Ratio
NLR	Negative Likelihood Ratio
DOR	Diagnostic Odds Ratio
PFI	Permutation Feature Importance
LIS	Laboratory Information Systems
EHR	Electronic Health Records
PCR	Polymerase Chain Reaction
XAI	Explainable Artificial Intelligence
